# Advances in Antiplatelet Therapy for Dentofacial Surgery Patients: Focus on Past and Present Strategies

**DOI:** 10.3390/ma12091524

**Published:** 2019-05-09

**Authors:** Gabriele Cervino, Luca Fiorillo, Ines Paola Monte, Rosa De Stefano, Luigi Laino, Salvatore Crimi, Alberto Bianchi, Alan Scott Herford, Antonio Biondi, Marco Cicciù

**Affiliations:** 1 Department of Biomedical and Dental Sciences and Morphological and Functional Imaging, Messina University, 98100 Messina ME, Italy; gcervino@unime.it (G.C.); lfiorillo@unime.it (L.F.); rsdestefano@libero.it (R.D.S.); 2 Multidisciplinary Department of Medical-Surgical and Odontostomatological Specialties, University of Campania “Luigi Vanvitelli”, 80121 Naples, Italy; luigi.laino@unicampania.it; 3 Department of General Surgery and Medical-Surgery Specialities, University of Catania, 95100 Catania CT, Italy; inemonte@unict.it (I.P.M.); torecrimi@gmail.com (S.C.); alberto.bianchi@unict.it (A.B.); antonio.biondi@unict.it (A.B.); 4 Department of Maxillofacial Surgery, Loma Linda University, Loma Linda, CA 92354, USA; aherford@llu.edu

**Keywords:** antiplatelet drugs, oral surgery, dental extraction, cardiovascular risk, dentofacial surgery

## Abstract

Background: Nowadays, patients involved in antiplatelet therapy required special attention during oral surgery procedures, due to the antiplatelet drugs assumption. The motivations of the assumption may be different and related to the patient’s different systemic condition. For this reason, accordingly to the current international guidelines, different protocols can be followed. The aim of this work is to analyze how the dentist’s approach to these patients has changed from the past to the present, evaluating the risk exposure for the patients. Methods: This review paper considered different published papers in literature through quoted scientific channels, going in search of “ancient” works in such a way as to highlight the differences in the protocols undertaken. The analyzed manuscripts are in the English language, taking into consideration reviews, case reports, and case series in such a way as to extrapolate a sufficient amount of data and for evaluating the past therapeutic approaches compared to those of today. Results: Colleagues in the past preferred to subject patients to substitution therapy with low molecular weight anticoagulants, by suspending antiplatelet agents to treatment patients, often for an arbitrary number of days. The new guidelines clarify everything, without highlighting an increased risk of bleeding during simple oral surgery in patients undergoing antiplatelet therapy. Conclusion: Either patients take these medications for different reasons, because of cardiovascular pathologies, recent cardiovascular events, or even for simple prevention, although the latest research shows that there is no decrease of cardiovascular accidents in patients who carry out preventive therapy. Surely, it will be at the expense of the doctor to assess the patient’s situation and risk according to the guidelines. For simple oral surgery, it is not necessary to stop therapy with antiplatelet agents because the risk of bleeding has not increased, and is localized to a post-extraction alveolus or to an implant preparation, compared to patients who do not carry out this therapy. From an analysis of the results it emerges that the substitutive therapy should no longer be performed and that it is possible to perform oral surgery safely in patients who take antiplatelet drugs, after a thorough medical history. Furthermore, by suspending therapy, we expose our patients to more serious risks, concerning their main pathology, where present.

## 1. Introduction

**Figure 1:**
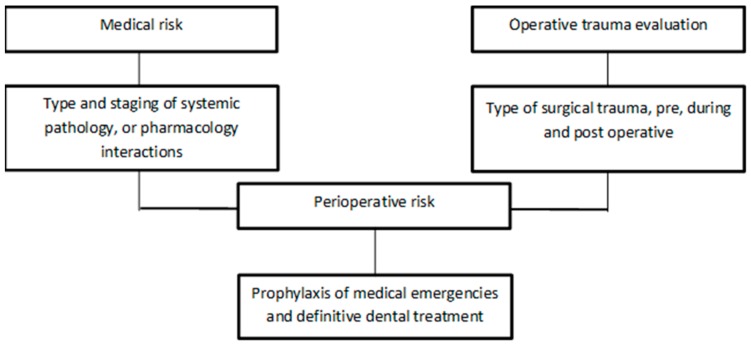
Cardiopatic patients risk flow chart.

For the dentist, the evaluation of the patient with systemic diseases tends to a summary diagnosis that has the function of reducing the risk of complications caused by dental intervention and to plan an appropriate therapy to the general condition of the patient. To this aim, multiple aspects of the problem must be compiled: highlight the patient’s own medical risk based on the type and severity of the disease; verify the patient’s suitability for outpatient therapy or the need for hospitalization; quantify the potential risk of dental intervention to choose the most correct treatment plan among the various possible alternatives; take the necessary precautions to prevent medical emergencies (Figure 1). Many patients are treated with antiplatelet drugs nowadays, as many go to the dental practices for surgical treatment [1]. A typical past approach was to suspend the therapy and to carry out a “substitutive” therapy with other anticoagulant drugs [2]. This type of approach has now passed and the literature informs us about the fact that the risk of bleeding in these cases, as bleeding would be circumscribed and easily tamponable, is minimal. Anti-platelet drugs can hinder platelet aggregation, reducing the risk of thrombus formation. Also known as, antiplatelet agents, these drugs are used in patients suffering from cardiovascular diseases or who are in particular conditions at risk for thrombus formation. The formation of the latter causes obstruction of the blood vessels (both venous and arterial) leading to more or less serious consequences, sometimes fatal, depends on the type of vessel that is affected by the obstruction. The currently available antiplatelet drugs are different and act through different mechanisms of action. As mentioned, antiplatelet drugs are used in all those patients suffering from cardiovascular disorders. More in detail, among the main indications of antiplatelet agents, we recall: Prevention of major atherosclombotic events after myocardial infarction or stroke, in patients with unstable angina pectoris, or with chronic stable angina pectoris; Prevention of cardiovascular events in patients with established atheromatous disease and in patients on hemodialysis; Prevention of thrombosis in patients undergoing extracorporeal circulation. Depending on the medicine used and the type of active ingredient contained, the therapeutic indications may be slightly different [3]. Logically a different story is for patients on oral anticoagulant therapy [4,5].

## 2. Materials and methods

### 2.1. Review Parameters

#### 2.1.1. Focus Questions

The main questions of this study is:

What is the current trend for patients, in antiplatelet therapy, who undergo oral surgery?

Are there updates in the literature regarding the pharmacological protocols in these patients, compared to the past?

#### 2.1.2. Information Sources

The search strategy incorporated examinations of electronic databases, supplemented by hand searches. A search of four electronic databases, including Ovid MEDLINE, PubMed, EMBASE, and Dentistry and Oral Sciences Source, human syndromes for relevant studies published in the English language to 201 was carried out. A hand search was also performed in other medical journals. The search was limited to English language articles. A hand search of the reference lists in the articles retrieved was carried out to source additional relevant publications and to improve the sensitivity of the search.

#### 2.1.3. Search

The following key-words were used: “antiplatelet drugs”and “dental extraction” or “Oral surgery”. The choice of keywords was intended to collect and to record as much relevant data as possible without relying on electronic means alone to refine the search results. The research was also limited to medical journals and only to articles written in English.

#### 2.1.4. Inclusion and Exclusion Criteria

The full text of all studies of possible relevance was obtained for assessment against the following inclusion criteria:

- Study of patients with antiplatelet therapy.
- Study of patients with dual or new antiplatelet drug therapy.

The applied exclusion criteria for studies were as follows:

- Studies involving patients with other specific diseases, immunologic disorders, uncontrolled diabetes mellitus, osteoporosis, or multi-therapy.
- Not enough information regarding the selected topic, no information about oral status and oral health or pharmacological therapy.
- No access to the title and abstract in English language or letters and editorials.
- Animal studies.
- Not full text articles.

#### 2.1.5. Risk of Bias Assessment

This type of work brings together all the studies in the literature in recent years presenting a review and a commentary about recent data and current trends during surgery in anti-aggregated patients. The risk of bias is minimal as the work is intended to be a collection of works carried out about these patients and about dental surgery, but we can only consider full text- and abstract-accessible articles in the English language. Regardless of the results of the studies taken into consideration, the evaluation was carried out by an adequate data analysis.

### 2.2. Type of Anti-Platelet Drugs

Platelet aggregation represents the final stage of the process that leads to the formation of the platelet cap and lays the foundations for the activation of the coagulation system, with activation of thrombin and the formation of the fibrin clot. The biochemical mechanism that allows activated platelets to bind together is represented by the exposure of the glycoprotein GPIIb-IIIa complexes, which act as fibrinogen receptors. Platelets are just some of the many actors involved in the coagulation process. Following the lesion of a blood vessel, the release of some chemicals by endothelial cells, and the exposure of the collagen of the damaged wall, determine the activation of platelets. The platelets rapidly adhere to the collagen exposed in the damaged wall and are activated releasing particular substances in the area of the lesion. These factors promote the activation and association of other platelets, which aggregate to form a fragile stopper, the so-called white thrombus; moreover, they contribute to reinforce the local vasoconstriction previously triggered by some paracrine substances, released by the injured endothelium with the aim of decreasing blood flow and pressure. Both reactions are mediated by the release of substances contained within some platelet granules, such as serotonin, calcium, ADP, and platelet activating factor (PAF). The latter triggers a signaling pathway that converts the phospholipids of the platelet membrane into thromboxane A2, which has a vasoconstrictor action and promotes platelet aggregation. Platelets are extremely fragile: a few seconds after the lesion of a vessel they aggregate and break, releasing the contents of their granules into the blood and favoring the formation of the clot. The aggregation of thrombocytes must obviously be limited to prevent the platelet plug from spreading into areas not affected by endothelial damage; platelet adhesion to healthy vessel walls is, thus, limited by the release of NO and prostacyclin (an eicosanoid). The primary platelet plug is consolidated in the next phase, in which a series of coagulocomplessively known reactions known as the coagulation cascade follow each other rapidly; at the end of this event the platelet plug is reinforced by a weave of protein fibers (fibrin) and is called a clot. Fibrin originates from a precursor substance, fibrinogen, thanks to the activity of the thrombin enzyme. While prostacyclin released by healthy endothelial cells inhibits platelet adhesion, our body synthesizes anticoagulants—such as heparin, antithrombin III, and protein C—to block and regulate certain reactions involved in the coagulation cascade, which must necessarily be confined to the injured area. Platelets have an essential role in stopping bleeding, but they do not intervene directly in the repair of the damaged vessel, which is, instead, due to cell growth and division processes (fibroblasts and smooth vascular muscle cells). Once the leak has been repaired the clot dissolves slowly and retracts due to the action of the enzyme plasmin trapped inside the clot (Figure 1).

The fibrinogen bound to adjacent platelet receptors forms real bridges between platelet and platelet and allows the formation of platelet aggregates. Fibrinogen is a blood plasma glycoprotein synthesized by the liver and endothelial tissue. The fibrinogen molecule is, in turn, composed of three simpler chains of amino acids, indicated as A-alpha, B-beta, and gamma, respectively. The mechanisms through which the fibrinolysis system is expressed are the following:

- Cellular mechanism: the white blood cells present near the clot release enzymatic substances capable of dissolving the clot; and
- Plasmatic mechanism: the fundamental step of this mechanism is the transformation of plasminogen into plasmin.

**Figure 2:**
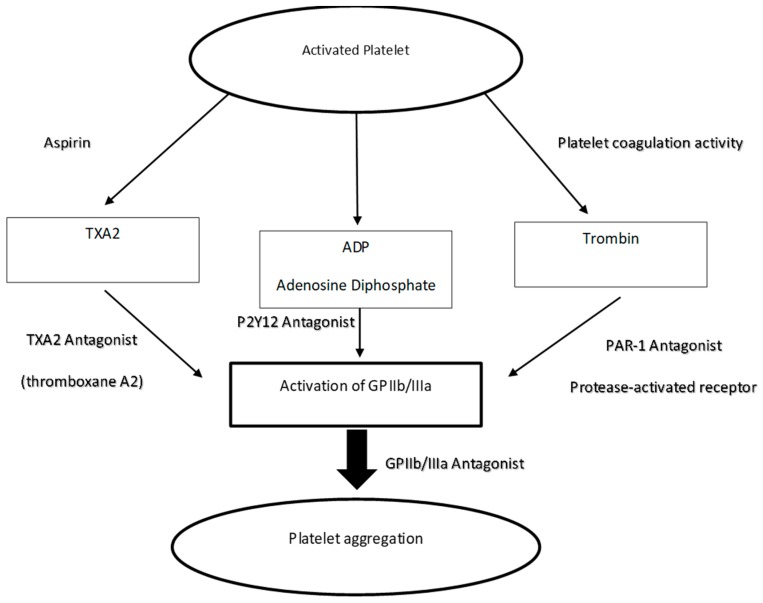
In the diagram the three ways that can inhibit platelet aggregation are evident. Antiplatelet drugs can act through three mechanisms: Interaction with platelet receptors for substances produced on the outside of platelets, such as collagen, thrombin, some prostacyclines and catecholamines. Interaction with platelet receptors for substances produced within platelets such as ADP, serotonin and prostaglandins D2 and E2. Interaction with platelet receptors for substances produced in platelets such as thromboxane A2, cAMP, cGMP and calcium ions [1].

Plasmin is the proteolytic enzyme that gives rise to the formation of degradation products of fibrinogen and fibrin (FDP), of which d-dimer is very important, routinely dosed in coagulation analysis: the reaction catalyzed by this enzyme is precisely the transformation of the insoluble clot fibrin into fibrin degradation products. Plasminogen activators promote fibrinolysis and, therefore, have an anti-coagulative effect. They may have intrinsic, exogenous, and extrinsic origins. Among the inhibitors of cyclooxygenase type 1 used as antiplatelet agents in the therapeutic field we find: acetylsalicylic acid, a known non-steroidal anti-inflammatory drug which has proved to be an excellent antiplatelet platelet when given to low doses of 75–300 mg; Indobucene; or Triflusal. To prevent the formation of thrombi, these antiplatelet drugs are administered orally. Acetylsalicylic acid and triflusal are irreversible inhibitors of the enzyme cyclooxygenase type 1 (COX-1), responsible for the synthesis of prostaglandins and, in particular, PGH2. From the PGH2 derive other prostaglandins and thromboxane A2 (TXA2). The latter is released following the degranulation of the platelets and, acting as second messenger, it recalls other platelets favoring the formation of the coagulum. By blocking the synthesis of TXA2 it is, therefore, possible to prevent platelet aggregation and, thus, the formation of the coagulum (Figure 2). Through long-term therapy with this type of drugs, a cumulative effect of platelet inactivation is achieved, since COX-1 is permanently inhibited and they, being denucleates (without nucleus), are unable to synthesize it again. Indobucene, on the other hand, acts as a reversible inhibitor of platelet COX-1; however, its efficacy is comparable to that of acetylsalicylic acid.

Among the type 3 phosphodiesterase inhibitors commonly used to hinder platelet aggregation and thrombus formation, we find: dipyridamole, which can be used by both only, both in association with acetylsalicylic acid; and cilostazol. Unlike other antiplatelet agents, it presents specific and exclusive therapeutic indications for the treatment of intermittent claudication. Both active ingredients are administered orally.

Another type of antiplatelet drug is a P2Y receptor antagonist. These are mainly prodrugs that, once transformed into the corresponding active metabolite, interact with the P2Y receptor whose endogenous substrate is represented by ADP. Among the active ingredients used in therapy, we find: ticlopidine; clopidogrel; ticagrelor; and prasugrel. As with PDE-3 inhibitors, some P2Y receptor antagonists can also be administered in combination with acetylsalicylic acid. Anti-platelet anti-inflammatory drugs of P2Y receptors are usually administered orally. As can be guessed from their own name, the antiplatelet drugs in question act as P2Y receptor antagonists, of which there are two forms: P2Y1 and P2Y12. The substrate of these receptors is represented by ADP (adenosine diphosphate), a nucleotide that plays a crucial role in the processes of platelet aggregation, therefore, in the formation of blood clots and thrombi. In detail, following the interaction of the ADP with the P2Y1 receptor, there is a change in the shape of the platelets. Following the interaction of ADP with the P2Y12 receptor, instead, there is a noticeable increase in platelet aggregation due to the inhibition of adenylate cyclase (enzyme used to convert ATP—adenosine triphosphate—into cAMP) a lowering of cAMP levels that we remember to be an inhibitor of platelet aggregation. Anti-platelet anti-agonizing agents of P2Y receptors are irreversibly bound to the P2Y12 form, thus hindering the link with the ADP and the consequent increase of the platelet aggregation that derives from it.

**Figure 3:**
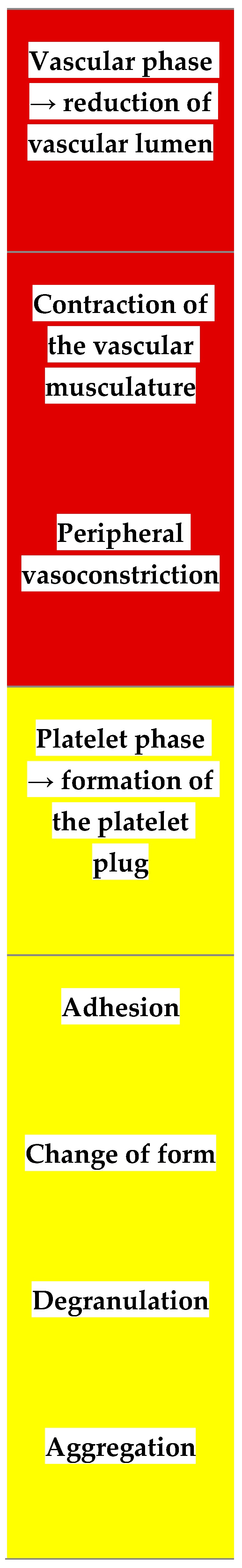

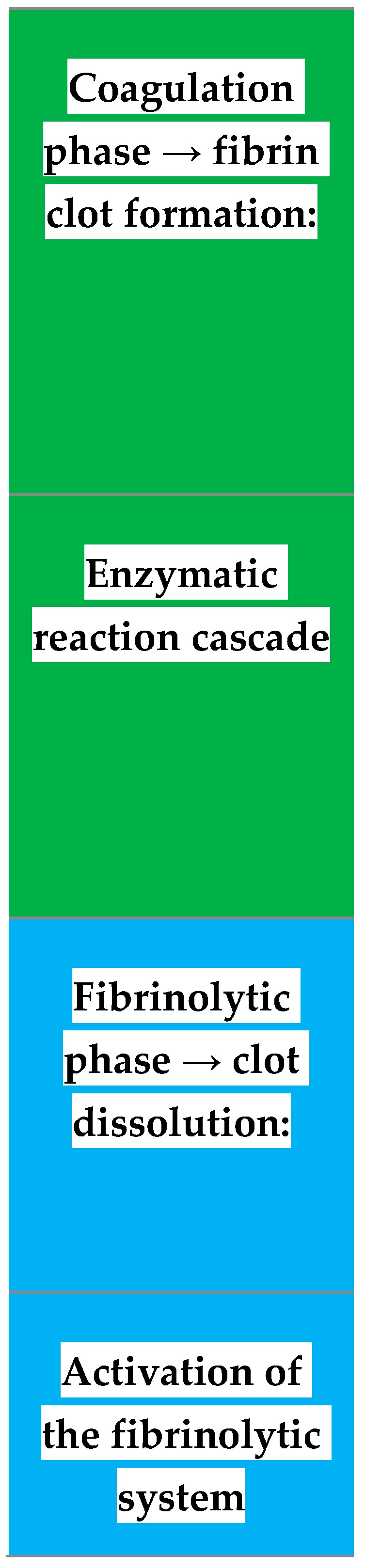
Hemostasis process phases.

Anti-platelet drugs with GP IIb/IIIa receptor antagonist action are used in the hospital setting and are given intravenously. The use of this type of antiplatelet agents is carried out in association with acetylsalicylic acid and unfractionated heparin for: Preventing myocardial infarction in the initial phase in patients suffering from unstable angina. The GP IIb/IIIa receptor is located at the platelet level; its natural substrate is fibrinogen. The GP IIb/IIIa antagonists prevent the binding with the fibrinogen, thus hindering the platelet aggregation, hence the formation of the blood clot and of the thrombus. The glycoprotein IIb/IIIa belongs to the family of integrins and is a heterodimeric membrane protein, present only on thrombocytes. During the blood coagulation process, then when the platelets are activated by thrombin, collagen, or thromboxane A2, the protein undergoes a conformational modification. This modification allows it to function as a receptor for the fibrinogen and for the von Willebrand factor, which anchor the platelets between them and on the extraneous surfaces. The inhibition of agonist binding to this receptor leads to blockage of platelet aggregation. The inhibitors of this receptor are, therefore, powerful antiplatelet agents. Eptifibatide is a cyclic peptide with a sequence analogous to the carboxy-terminal end of the fibrinogen delta chain, which mediates the binding of fibrinogen to its αIIIbβ3 receptor. It is administered as a bolus followed by an infusion for 96 hours. It is used to treat acute coronary syndrome and in the case of coronary angioplasty. Tirofiban is a non-peptide that appears to have an effect and efficacy similar to Eptifibatide (cases of death and infarction reduced by 20% compared to placebo). It is effective in myocardial infarction without Q wave and unstable angina. Abciximab is a chimeric monoclonal antibody, the first approved drug of this class. It also acts on the vitronectin receptor on platelets, vascular endothelial and smooth muscle cells. It is used in patients undergoing percutaneous angioplasty for coronary thrombosis and acute coronary syndrome. When administered with aspirin and heparin, it is effective in the prevention of restenosis, and in recurrent myocardial infarction (Figure 3).

Thienopyridine drugs are, therefore, a category of antiplatelet agents able to inhibit the activity of P2Y12 and, therefore, reduce platelet activation. The first synthesized drug belonging to this category was ticlopidine; a second-generation thienopyridine, on the other hand, is clopidogrel, today widely used in the secondary therapy of acute coronary syndromes in association with aspirin; a third-generation thienopyridine, prasugrel, is already commercially available in the United States. Then there are ongoing studies on two “non-thienopyridine” molecules, that is, with a different molecular structure, which are also able to inhibit the activity of P2Y12, cangrelor and ticagrelor. Although both PAR-1 and PAR-4 are able to induce platelet secretion and aggregation, the interest has been almost exclusively directed to PAR-1 because this receptor is capable of activating platelets at thrombin concentrations a hundred times less than PAR-4. Vorapaxar (synthetic tricyclic 3-phenylpyridine), is a non-peptide competitive antagonist of PAR-1 characterized by high selectivity and low molecular weight. Due to its specificity it does not hinder the aggregation induced by other agents such as ADP, arachidonic acid, or collagen and, therefore, does not prevent the intervention of platelets in hemostasis (Figure 2).

Among the anti-platelet agents of the thromboxane A2 (TXA2) receptor and of the synthesis of the same we find the picotamide. It is an orally-administered active substance that is able to carry out its antiplatelet activity through two mechanisms of action: Inhibits thromboxane-synthase (enzyme that converts PGH2 to TXA2), thus preventing the synthesis of TXA2. It blocks the TXA2 platelet receptors, hindering platelet aggregation (Table 1) [6,7,8].

#### 2.2.1. Past Protocols Collection

As mentioned above by a careful analysis of the past literature, the routine in the case of patients with antiplatelet therapy, provided for the suspension of the therapy. Even some protocols, to be printed to patients, included warnings of this type:

In case of incontrollable bleeding:

- you should contact the following phone number…; or
- in case of absence, do not hesitate to show up at the hospital emergency service (public, private), or consult your own physician [1].

The old protocols predicted, on the basis of the patient’s pathology, the simple suspension of therapy with antiplatelet agents (APA), or the replacement of the latter with other drugs. Before undertaking any dental operation or oral surgery it was mandatory and logical to suspend this type of therapy from what was extracted from the literature. For many years, therefore, this type of therapy was suspended before dental treatment, which is unacceptable today as the suspension of these treatments for 1–2–3 weeks exposes the patient to a much higher thromboembolic risk than the bleeding that it can derive from it. Moreover, the suspension of these drugs for arbitrary times, such as 4–5, days is completely useless and without advantages, except the single thromboembolic risk (Table 2) [9].

#### 2.2.2. Today’s Protocols

Thrombocytopathy may result in prolonged bleeding following oral surgery which generally presents as immediate, not serious, bleeding, and controllable with local hemostatic measures. The effect may vary depending on the individual subject, type of drug, dosage used, and duration of therapy. Local hemostatic measures are sufficient in the absence of other competing factors (Table 3). The association of analgesics and anti-inflammatory agents should be avoided to interfere with hemostasis; safe drugs resulted in noramidipirine, paracetamol, and narcotics; among the relatively safe Fans are naproxen and ibuprofen; nimesulide does not alter platelet activity in vivo significantly. Simple interventions can be performed without requiring laboratory tests and medical advice; it is sufficient to reduce the surgical trauma (correct incision and disconnect) and measures of local hemostasis such as ice, compression, topical hemostats, and for rinses (tranexamic acid).

In case complex operations are required, characterized by a high risk of bleeding, it is advisable to plan the intervention and, after medical advice, rather than replacing or suspending therapy, in today’s protocols it is preferable to focus attention on something else than the patient’s main pathology. The factors taken into consideration are the reasons why our patients perform such prophylaxis or therapy. The attention then shifts to the management of the anxiety of our patient, to reduce the cardiovascular risk, and still, in the case of surgical treatments, on the careful management of pain and anesthetic drugs, with adrenaline or without, considering also the lower efficiency of these and also stressing the risk/benefit factor preferring the administration of anesthetics with adrenaline as more effective to control pain. The results suggest that continuing the intake of antiplatelet drugs did not increase bleeding after placement of dental implants, and patients taking aspirin, clopidogrel, ticagrelor, and dual antiplatelet therapy experienced acceptable rates of controllable bleeding [9,10,11]. Many protocols provide consultation by a cardiologist or a hematologist; in any case there are maneuvers to control the bleeding of these patients as we remember that they are not coagulated. From a point of view of oral surgical practice, there are a whole series of maneuvers to limit or block any bleeding, maneuvers that, in cases of patients like these, should be attentively attended by the clinician as necessary. The healing of a post-extraction alveolus can occur as a second intention as we verify daily. In these cases, using devices such as a fibrin sponge or a fibrin cone inside the post-extraction socket can be an excellent aid. Additionally, use of sutures to avoid the destruction of coagulation caused by mechanical causes or saliva itself is very useful, and cross-mattress sutures are very compact and allow keeping the material positioned or the coagulum itself in place even if, from the point of view of the flaps, we know that it is not possible to get an approach in these cases. In the case of flap surgery, the closure must take place in a meticulous way, making sure not to have bleeding points or mobile flaps. In spite of the bleeding time, therefore, these drugs are increased to allow oral surgery, advising the use of local hemostats [12,13].

The current European protocols, most widely accepted, are those proposed by the European Society of Cardiology in the Focused Update on Dual Antiplatelet Therapy (DAPT). The guidelines recommend performing a practical and sequential assessment of the patient according to both clinical risk factors and the results of instrumental investigations and the stress induced by the surgical act, so as to obtain a personalized risk estimate and to select the therapy, coronary intervention, or surgical and anesthesiological techniques most suitable for optimizing the patient’s condition in the perioperative phase. The proposed guidelines certainly take into consideration, first, a preoperative evaluation, an evaluation that must carefully evaluate the risk of cardiac events in relation to the surgical procedure and all related factors, such as fluid loss, changes in body temperature, bleeding, duration of surgery, and related stress. It is, therefore, possible to perform a risk analysis related to the intervention, evaluating some factors, such as cardiac function, the evaluation of biomarkers, and the carrying out of non-invasive (electrocardiogram) or invasive (angiography) examinations. It is, therefore, possible to implement strategies to reduce the risk, and implement pharmacological strategies, such as with beta-blocking drugs, statins, nitrates, calcium antagonists, or angiotensin-converting enzyme inhibitors.

Antiplatelet agents, anticoagulants, and diuretics can represent other pharmacological prophylactic therapies. For example, the use of aspirin was found to be widely effective in preventing intra and postoperative stroke in patients with coronary artery stents (ACS). According to the guidelines of the European Society of Cardiology, often for fear of bleeding the therapy is replaced or suspended, but the risk of bleeding is very low compared to the patient’s cardiological risk. Patients with a drug-eluting stent (DES) can be treated 12 months after implantation of the stent, and a double anti-platelet therapy is recommended on them during surgery. In the case of the metallic stent, on the other hand, surgical therapy can be performed starting from six weeks after implantation, always with double anti-platelet therapy for at least six weeks, and preferably three months. The same guidelines cited propose a 6-step protocol with a YES/NO algorithm to follow to identify and plan the surgery in patients at cardiovascular risk. From assessing the urgency of the surgical procedure (step 1), analyzing any patient’s cardiac instability (step 2), defining the risk of the surgical procedure (step 3), and considering the patient’s functional capacity (step 4), to evaluate the pharmacological therapy of the patient, recommending to continue the therapy (step 5), and in patients with moderate or poor functional capacity to evaluate the risk related to the procedure and the type of patient (step 6).

### 2.3. Type of Pathology That Needs This Therapy

Antiplatelet drugs are approved for use during percutaneous coronary intervention and in acute coronary syndromes. Aspirin at 325 mg/day is used in secondary prevention in subjects with a history of vascular accidents. In a study that earned the 1982 Nobel Prize for medicine, the mechanisms of the human organism that block the production of prostaglandins and thromboxanes by enzymatic inhibition were demonstrated. The inhibition occurs because the cyclooxygenase enzyme, involved in their synthesis, is irreversibly acetylated by one addictive reaction acetylsalicylic acid, making it no longer functional. Prostaglandins are local hormones produced by the body and perform a variety of functions, among which includes the transmission of the pain signal to the brain and the modulation of body temperature at the hypothalamus level. The thromboxanes are involved in the process of blood coagulation and are, therefore, essential for hemostasis. Heart attacks are mainly due to the obstruction of blood vessels by clots of coagulated blood. The use of a small amount of aspirin daily leads to a reduction in the number of clots [14].

In primary prevention, that is, in people who have not had cardiovascular events, it is not currently recommended, because, in the face of a reduction of non-fatal myocardial infarction, the intake does not lead to a reduction in total cardiovascular death, but instead increases clinically important bleeding, or even following invasive medical treatment. It should be remembered that the most important side effect is a lower blood capacity to coagulate, which results in more abundant bleeding in certain situations, which may also be related to the well-known gastrolysis of the drug. Aspirin inhibits cyclooxygenases 1 and 2. COX-1 is present in the platelets and, being acetylated, undergoes an irreversible inactivation; COX-2 is mainly found in endothelial cells and, as these latter are provided with nuclei, neosynthesis is possible. The use of aspirin, therefore, inhibits the formation of thromboxanes by COX-1, while the synthesis of prostaglandins and prostacyclines is restored fairly rapidly, shifting the thrombotic balance towards platelet anti-aggregation. Aspirin is, therefore, normally used in thrombotic disorders as an antiplatelet and is called Cardioaspirin. New non-steroidal anti-inflammatory drugs, called “selective COX-2 inhibitors” (“Coxib”) have been developed, with the hope that they present even more reduced side effects on the gastro-intestinal system. It has been shown that the reduction of gastrotoxicity is marked. On the other hand, with COX-2 inhibited, the thromboxane synthesis of the platelets increases, through COX-1 is still active. This shifts the balance toward thrombotic platelet aggregation, favoring the formation of clots and the onset of serious cardiovascular pathologies [15].

Clopidogrel and ticlodipine are used in coronary units in patients with acute myocardial infarction and unstable angina. Dipyridamole may be associated with aspirin for the prevention of secondary vascular events. Cilostazol is instead approved in the therapy of intermittent claudication. The complication of internal bleeding with debilitating or fatal outcomes derived from the two principles of acetylsalicylic acid (ASA), clopidogel, and vitamin K antagonists have been analyzed by two large independent studies, both on more than 30 hospitalized patients following an initial myocardial infarction, showing that the risk, in increasing order, is: ASA only, ASA-clopidogel association, vitamin K antagonists/ASA, and vitamin K antagonist/clopidrogel. Assumptions of the three drugs are as follows: meta-analysis until October 2010, on 100,000 patients with diabetes and low-risk diseases (75–325 mg/day) without cardiovascular disease: 30% increase risk of debilitating or fatal bleeding, and more gastrointestinal, reduced proton pump inhibitors [6,16]. In addition to primary or preventive reasons, it is possible that patients who carry out this type of therapy may come to our attention because they have had cardiovascular events in the past. Among these we certainly recognize the acute coronary syndromes (ACS) and patients undergoing stents, whether these are indicated, or not, as bare metal stents (BMS) or drug-eluting stents (DES).

#### 2.3.1. Type of Oral Surgery Intervention That May Have a Bleeding Risk

**Figure 4:**
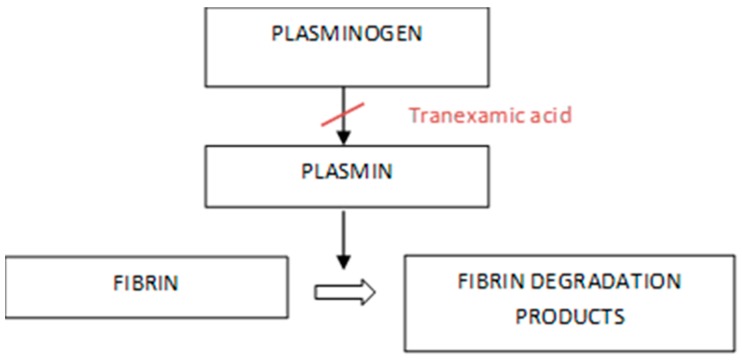
Tranexamic acid working [1].

In the dental field there are many treatments that can expose the patient to a risk of bleeding. Surely the patient not on antiplatelet therapy is not a patient to be considered at as much a risk as the patient on anticoagulant therapy. In dentistry some performances, even if they foresee bleeding, have no risk for the patient, not even for the coagulated one. For example, some of these procedures are conservative or non-surgical endodontic therapeutic ones. Another topic is oral hygiene, such as scaling, which, in some cases, can cause significant bleeding. In some of these cases it is also necessary to consider the transient bacteremia occurring during these treatments and, therefore, the risk of bacterial endocarditis if the patient is in therapy for cardiopathies [17]. Dental therapies that cause bleeding cause a transient bacteremia quantitatively proportional to the local trauma and local inflammation; some bacteria (*Streptococcus viridans* and *Staphylococcus aureus* are more frequent) can colonize the platelet vegetations present on presumptive valve lesions. It is believed that 1:5 cases of subacute endocarditis is associated with dental therapies and that, in most cases, the disease appears within two weeks of surgery [18,19]. In oral surgery, thrombocytopathy can cause prolonged bleeding, which usually occurs as an immediate, not serious, bleeding, controllable with local hemostatic measures. The effect may vary depending on the individual subject, type of drug, dosage used and duration of therapy. After surgery, the association of analgesics and anti-inflammatory agents that interfere with hemostasis must be avoided; safe drugs are noramidipirine, paracetamol, and narcotics; relatively safe are naproxen and ibuprofen; and nimesulide does not alter platelet activity in vivo significantly [20]. Simple interventions can be performed without requiring laboratory tests and medical advice. It is sufficient to reduce the surgical trauma (correct incision) and measures of local hemostasis, such as ice, compression, topical hemostats, and for rinses (tranexamic acid). If complex interventions are necessary, characterized by a high risk of bleeding, it is advisable to program the surgery and, after medical advice, stop the administration of antiplatelets to allow normalization of the time of bleeding [20,21,22]. In case of emergency surgery, the risk of bleeding may be reduced by administering desmopressin nasal spray or intravenous infusion. An intraoperative and postoperative method of local or systemic prophylaxis is represented by tranexamic acid [23,24]. This is an antidote for fibrinolytics and is an excellent method for post-operative hemorrhages in the dental field. This can be administered locally, using a sterile gauze or intravenously with slow infusion. Due to the almost exclusively renal elimination of the substance, in renal impairment the dose should be reduced, especially in prolonged use, to prevent the accumulation of tranexamic acid in the plasma (Figure 4). The number of single doses per day is reduced depending on serum creatinine levels. The mechanism of action is based on a blockade of plasmin formation, through the inhibition of the proteolytic activity of plasminogen activators, ultimately resulting in an inhibition of blood clot lysis. Surgical operations are performed under local anesthesia, which often presents different dosages of vasoconstrictors in dental formulations. A reduced tolerance to anesthetics is present in hepatopathies that lead to a decreased metabolism of amide anesthetics; in genetic deficits of blood pseudocholinesterases that decrease the metabolism of esthetic anesthetics. The pharmacological interactions between anesthetic and other drugs administered for systemic disorders are represented by:

- Depressors of the central nervous system (alcohol, antidepressants, sedatives—-respiratory and CNS depression;
- Antiarrhythmics—cardiac depression;
- Antimiastenics—antagonism of the antimyasthenic effect; and
- Beta-blockers—prolonged anesthetic effect.

A particular effect is attributable to prilocaine, which causes a potentially dangerous methemoglobinemia in patients with hereditary methemoglobinemia. Systemic diseases that result in reduced tolerability to vasoconstrictors result in heart disease, arterial hypertension, and glaucoma. In patients with cardiovascular disease, 0.036 mg norepinephrine (two vials of 2% lidocaine with noreprinephrine 1:100,000) or equivalent is considered to be advantageously used (profound and prolonged anesthesia, reduction of bleeding) greater than the risk of cardiac work overload represented by the adrenergic reaction triggered by pain due to insufficient anesthesia [25]. The laboratory tests in these cases, useful for the clinician, are different. With the “vascular fragility test”, the systolic and diastolic arterial pressure is determined and the sphygmomanometer cuff is inflated for 5 min at an intermediate value. The appearance of petechiae on the arm distal to the cuff is positive for a non-specific alteration of hemostasis (platelet, vascular, coagulation pathology). The “bleeding time” is the only in vivo test able to explore the interaction between platelets and the vessel wall and, therefore, to evaluate the first stages of the hemostatic process (primary hemostasis). There are several method for measuring primary hemostasis of PFA-100 accordingly to Favaloro et al. [26]. Therefore, Simurda et al. documented in their published paper a patient with von Willebrand disease type III, affected by prolonged bleeding time and extreme prolonged examination of PFA-100 Col/ADP and PFA-100 Col/Epi (>300 s) [27]. However, the most commonly used standard technique is derived from the Mielke method (standard incisions 5 mm in length and 1 mm in depth under standard venous pressure conditions of 40 mm Hg obtained by inflating the arm of a sphygmomanometer). The test is elongated in case of quantitative and/or qualitative alterations of the platelets or their inadequate interaction with the vascular wall (e.g., M. of Von Willebrand) or, finally, for particular vascular anomalies. A normal bleeding time ensures that there is no clinically relevant platelet disorder in the test subject. The “platelet count” shows thrombocytopenia but does not provide indications on platelet function (thrombocytopathy due to aggregation deficits or adhesiveness caused by antiplatelet agents or other pathologies). The “prothrombin” or “quick time” investigates the functionality of the extrinsic and common coagulation pathway; it will be lengthened in the presence of factor VII deficiency and/or common path factors (V, X, prothrombin, fibrinogen). There are two other basic hemostasis tests—thrombin time and fibrinogen function—that need to be investigated in patients as part of screening tests. These tests follow the final part of hemostasis. They are prolonged in fibrinogen disorders accordingly to Simurda and Puetz [28,29]. The parameter can be expressed in three ways, the values of which are not strictly superimposable: in time (seconds), in international units INR (international normalized ratio), and in the percentage rate of residual prothrombin activity. Finally, partial thromboplastin time (APTT) studies all coagulation factors that contribute to the generation of thrombin, except factor VII. The normal value is 30–45 s and the security area is between 40 and 50 s. Time should not deviate more than 8–10 s [30].

#### 2.3.2. Primary or Secondary Pharmacological Therapy? Management of Anxiety in Cardiac Patients

We must always consider that patients undergoing antiplatelet therapy can be in primary or preventive therapy, or secondary to a past cardiovascular event. The apprehension, anxiety, and pain caused by the dental intervention provoke in the patient an alert reaction with activation of the sympathetic and parasympathetic systems (stress). The complex alterations that follow are proportional to the subject’s emotion: 80% of patients are able to control their emotions (like fear or anxiety); 15% develop an anxious reaction; 5% of patients develop an odontophobia that generates serious psychic conflicts. The functional overload imposed on the cardiovascular system can overcome the ability to adapt and precipitate some medical emergencies particularly in those with reduced functional reserve resulting from cardiovascular diseases. The symptoms of anxiety can be very varied and include: psychomotor agitation, sweating, loquacity, nervousness, cardiovascular and pressure changes. Stress can precipitate different types of reactions that in addition to specific complications, can decompensate any associated cardiovascular diseases (ischemic myocardiopathy, heart failure). The adrenergic reaction (hypersymaticotonia) is caused by the hypersecretion of endogenous adrenaline and is characterized by tachycardia and arterial hypertension. The vagal reaction (vagotonia) is characterized by pallor, nausea, arterial hypotension, bradycardia, lipothymia, or syncope. An epileptic seizure can be triggered in patients with epilepsy. Therefore, it is necessary to treat these patients with the appropriate tools, which are not always drugs or surgical techniques [31,32].

## 3. Results

The results of the individual articles in the research were carefully evaluated. The aim is to highlight actual bleeding risks in our patients. Patients who undergo antiplatelet therapy, however, do not have a greater risk of going against bleeding, in some cases additional hemostatic maneuvers are suggested and in others not. However, under no circumstances should the antiplatelet therapy of our patients carried out for their primary pathology or for prevention be changed or stopped.

## 4. Discussion

As already mentioned in the previous paragraphs, the approach of the clinicians toward these patients is highly variable, and has been even more so in past years. Now, however, the trend seems to change towards a more secure therapeutic strategy towards our patients. Tabrizi et al. in 2018, evaluated the cessation of the continuation of antiplatelet therapy in patients undergoing implant surgery. The study is split-mouth type and provides for the placement of two implants for each of the 22 patients. The two groups of patients take ASA 80 mg and clopidogrel 75 mg. The results of this study suggest that continued therapy does not increase the risk of bleeding after and during surgery [33]. Doganay et al., in 2018, assessed the risk of bleeding in patients taking ticagrelor. In addition, in this case the risk of postoperative bleeding was declared minimum or acceptable, in patients undergoing dental extractions or small oral surgery maneuvers [34,35], according to the authors, patients can undergo oral surgery safely. Patients undergoing oral surgery in the Rocha et al. 2018, study did not change their antithrombotic therapy, as this is not a risk factor for the patient [36].

Post-exodontia bleeding is also evaluated in the study of Lillis et al., 2017, with the aim of understanding the behavior of clinicians [37]. Behavior in clinical practice that often tends to not reflect international protocols, as previously mentioned in this article, is unfortunately highly variable. In this article there is no clinical information. Saez-Alcaide et al. in 2017 talked about the management of the patient in antiplatelet therapy, making a review on the behavior of dentists, and show that the current trend is to maintain their drug therapy [38]. A study by Sharma et al. evaluated the function of a hemostatic biomaterial after extractions on patients on antiplatelet therapy with a 2017 article. This agent had excellent hemostatic results, lowering both bleeding time and wound healing and, still, postoperative pain, without the need for other drug therapy, compared to the control group [31,39]. A case-crossover study by Akhlaghi et al. in 2017 assessed whether there is indeed a higher risk in these patients who underwent surgery. However, the result was that dental extractions could be addressed without modifying aspirin or clopidogrel therapy, despite some differences between the groups assessed in platelet reactivity and clot formation [40]. The bleeding was measured in patients by performing dual antiplatelet therapy after oral surgery by Medeiros et al., and in patients with dual antiplatelet therapy no complications were noted, so it was not necessary to stop the treatment [41].

**Figure 5:**
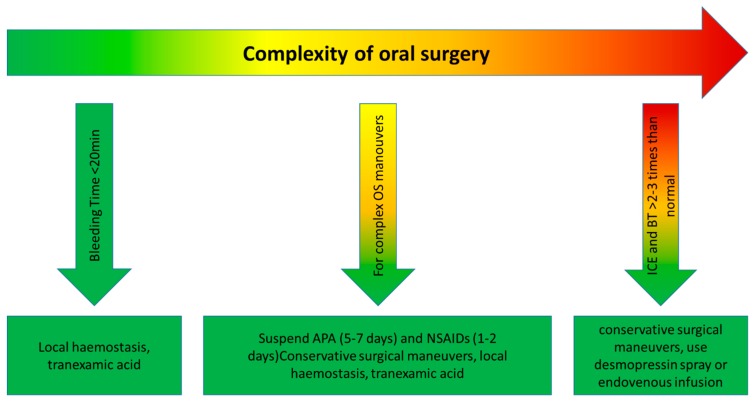
Protocol in patients on antiplatelet therapy related to the complexity of oral surgery (BT: bleeding time; ICE: in case of emergency; OS: oral surgery; APA: anti-platelet; NSAIDs: non-steroidal anti-inflammatory drugs).

Nagao et al. claim that POCT (point of care testing) may be useful for assessing the risk of bleeding in these patients [42]. Yanamoto et al. in 2017 carried out a retrospective multicentric study on the risk factors of hemorrhage after dental extractions. In patients on dual antiplatelet therapy, according to the authors, they are more likely to have a hemorrhagic risk, so local hemostasis techniques are recommended, such as sutures, gauze, and compression [43], or other more complex techniques, such as membrane flaps or grafts, biomaterials, or socket preservation [44,45], especially in case of oral inflammation [46,47]. Chee in his 2013 article already downloads the responsibility of the dentist to other medical colleagues. According to this article, in fact, patients on antiplatelet with a recent coronary artery stenting shoud be referred to their primary cardiologist on the cessation of these agents before any surgery [38]. In the latest articles taken into consideration, surgery and antiplatelet therapy are much debated. The dentist, even in patients undergoing therapy, can perform routine extraction without risk without interrupting or modifying the latter. Patients do not have a greater risk of bleeding, even if they have been in antiplatelet therapy for a long time but, in some cases, as some authors mention in a refined way, the therapy must be personalized for each patient [26,27,28,29,30,31,32,33,34,35,36,37,38,39,40,41,42] Pototski et al. in 2007, stated in their work that patients can be treated without risk if their INR is lower than 4.0 [48]. However, other studies assess that the risk of bleeding can be controlled by post-extraction hemostasis maneuvers [49]. Daniel et al., in 2002, discuss the risk of bleeding patients in antiplatelet therapy in an excellent scientific article, highlighting that the decision is based on an understanding of the pharmacodynamics of these drugs [50] and a thorough medical history of the patient. In addition, some syndromic patients will certainly respond to therapy differently and should be carefully evaluated [50,51,52,53,54,55,56]. Despite what has been previously mentioned it is, however, possible to highlight more complex cases [57,58,59,60,61,62,63,64,65,66,67,68,69,70] in which different pathologies coexist, or patients carrying out pharmacological polytherapies. In any case it is always necessary to evaluate the most recent and updated guidelines [71,72,73,74]. Conflicting information emerges from the literature, but most authors agree that the risk of emanation and general health is minimal for patients who undertake antiplatelet therapy (Figure 5).

## 5. Conclusions

In conclusion, with this work we have tried to give an account of the current situation from a clinical point of view. In the past, colleagues tended to perform replacement therapies or to arbitrarily suspend drug therapies, thus exposing patients to a high thromboembolic risk. Today the new trend does not change pharmacological therapies and intervenes conscientiously on patients. This article is intended for reference only to patients who have to undergo oral surgery and are in antiplatelet therapy, and does not want to deliberately include all the notions concerning the cardiovascular risk of the patient, such as rheumatic heart disease, congenital heart disease, and acquired valvulopathy, cardiac surgery, bacterial endocarditis, patients with cardiac arrhythmias, heart failure, ischemic heart disease, arterial hypertension, acute arterial hypotension, arteriosclerosis, anxiety, and anticoagulant therapy. The new guidelines are followed by dentists in the case of small surgery maneuvers. We hope that this article can be a guideline, and reach those colleagues who still follow old standards no longer recognized, which, as a sole result, can harm the patient and lead the clinician into legal-medical disputes.

## Figures and Tables

**Table 1:** Most common antiplatelet drugs with clinical indications and adverse effects.

***Cox 1 Inhibitor***	***Clinical Indication***	***Adverse Effect***
Aspirin	Ischemic stroke reduction risk, TIA, stable angina	Increased risk for gastrointestinal bleeding or hemorrhagic stroke
***ADP Receptor Blockers***	***Clinical Indication***	***Adverse Effect***
Clopidogrel	Stroke or percutaneous coronary intervention (PCI) or acute coronary syndrome (ACS)	Bleeding, abdominal pain, diarrhea, rash
Prasugrel	PCI or ACS	Risk of intracranial bleeding, diarrhea, nausea
Ticagrelor	PCI or ACS	Bleeding, dyspnea
Ticlopidine	Risk reduction for stroke in patients intolerant of aspirin	Thrombocytopenia, neutropenia
Cangrelor	PCI or ACS	Bleeding
***PDE Inhibitors***	***Clinical Indication***	***Adverse Effect***
Dipyridamole	After heart valve replacement	Headache, nausea, vomiting
***GP IIb/IIIa Inhibitors***	***Clinical Indication***	***Adverse Effect***
Abciximab	PCI or ACS	Excessive bleeding, including gastrointestinal or urinary tracts
Tirofiban	ACS	Bleeding, hematuria, thrombocytopenia, nausea, vomit, allergic reactions
Eptifibatide	ACS	Bleeding, hematuria, hypotension, thrombocytopenia, nausea, vomit, allergic reactions
***PAR-1 Inhibitors***	***Clinical Indication***	***Adverse Effect***
Vorapaxar	***ACS***	Anemia, bleeding, bruising, hematomas, gastritis, hematuria.

**Table 2:** Past protocol references.

*Past Protocols Literature*	*Year of Pubblication*	*in Case of Oral Surgery*
Terezhalmy et al.	1996	Supension of APA therapy
Allen et al.	1967	Supension of APA therapy

**Table 3:** Studies’ results.

*Author*	*Year*	*Results*
Tabrizi et al. [30]	2018	Continuing the intake of antiplatelet drugs did no increase bleeding after implant placement
Doganay et al. [31]	2018	Acceptable rates of bleeding after tooth extraction or minor oral surgical procedures
Rocha et al. [32]	2018	Dental surgery might be carried out without altering the regiment because of low risk
Lillis et al. [33]	2017	This article is an overview of oral surgery in cardiovascular patients
Sàez-Alcaide [34]	2017	The current trend is to maintain treatment
Sharma et al. [35]	2017	Use of hemostatic agents lessens the bleeding time
Akhlaghi et al. [36]	2017	Dental extraction can be performed safely without withdrawal of aspirin or clopidogrel
Medeiros et al. [37]	2017	There was no postoperative bleeding complication in any case
Nagao et al. [38]	2017	Use of tool for bleeding prediction can be safer
Yanamoto et al. [39]	2017	The risk of hemorrhage after tooth extraction is increased in dual therapy patients, use local hemostatic treatments
Chee et al. [40]	2013	Patients with recent coronary artery stenting should be referred to their primary cardiologist before any surgery
Koskinas et al. [41]	2012	Decision making concerning dental management of antiplatelet-receiving patients will need to be individualized and risk-tailored, choosing between the Scylla of local bleeding and the Charybdis of thrombosis in high-risk patients.
Al-Harkan et al. [42]	2012	Use local measure for hemostasis
Krishnan et al. [43]	2008	Routine dental extractions can be safely performed
Pototski et al. [44]	2007	Minor oral surgeries, biopsies, extraction or periodontal surgery can be safely be done
Garnier et al. [45]	2007	Hemorrhagic risk can be controlled by local hemostasis protocol
Daniel et al. [46]	2002	Could be useful an algorithm for decision making in these patients
